# Recent Advances in Genetics of Moyamoya Disease: Insights into the Different Pathogenic Pathways

**DOI:** 10.3390/ijms26115241

**Published:** 2025-05-29

**Authors:** Guangsong Han, Ming Yao, Jun Ni

**Affiliations:** Department of Neurology, Peking Union Medical College Hospital, Peking Union Medical College and Chinese Academy of Medical Sciences, Beijing 100730, China; b2023001167@student.pumc.edu.cn

**Keywords:** moyamoya disease, moyamoya syndrome, genetics, pathogenic, pathways

## Abstract

Moyamoya disease (MMD) is a rare yet clinically significant cerebrovascular disorder characterized by progressive stenosis of the distal internal carotid artery and/or its principal branches, accompanied by the development of characteristic collateral vessel networks. This disease demonstrates a complex multifactorial etiology with strong genetic determinants, as evidenced by its distinct geographical distribution patterns and familial clustering. Recent genetic researches have identified multiple pathogenic mutations contributing to MMD development through three principal mechanisms: progressive vascular stenosis, abnormal angiogenesis, and dysregulated inflammatory responses. Furthermore, moyamoya syndrome frequently occurs as a secondary vascular complication in various monogenic disorders. This review provides a comprehensive analysis of recent genetic advances in MMD in view of diverse pathogenic pathways, offering valuable perspectives on the molecular mechanisms underlying disease development and potential therapeutic targets.

## 1. Introduction

Moyamoya disease (MMD) is a rare and devastating cerebrovascular disease characterized by progressive stenosis of the distal internal carotid artery and/or its major branches, accompanied by the development of collateral vessel networks resembling a “puff of smoke” on angiography [1,2]. When these characteristic vascular manifestations coexist with identifiable risk factors or diseases (e.g., neurofibromatosis type 1, sickle cell disease), the entity is classified as moyamoya syndrome (MMS) [2,3]. Although the exact pathophysiology of MMD still remains incompletely understood, MMD is increasingly recognized as a polygenic disorder with environmental interactions [4]. The higher prevalence of MMD in East Asia compared to non-East Asia and the obvious familial aggregation highlight the significant role of genetic factors involved in the pathogenesis of this disease [3,4]. Advances in molecular genetics, including genome-wide linkage analyses, candidate gene association studies, and next-generation sequencing, have identified various linked chromosomal regions and susceptibility genes associated with MMD and these findings have been partially validated by functional research, which suggests that the pathogenesis of MMD cannot be explained by a single gene mutation but is more likely the result of combined effects from multiple genetic factors [4,5]. In aggregate, specific mutations linked to MMD mainly involve the following two mechanisms derived from the pathological and genetic studies: progressive vascular stenosis/occlusion secondary to intimal thickening, media attenuation and internal elastic lamina damage; aberrant angiogenesis manifested as the formation of moyamoya collateral vessels [5,6]. Emerging evidence further implicates that mutations associated with autoimmune and inflammatory responses are also important susceptibility factors in the development of MMD [7]. Furthermore, several congenital diseases can exhibit with moyamoya syndrome and the molecular pathways implicated in their pathogenic genes may offer new insights into the genetic basis of MMD [6].

In this review, we systematically summarized current research findings on the genetics of MMD through the lens of the above pathways. This synthesis specifically concentrates on high-impact studies from the past five years, incorporating novel findings from next-generation sequencing, genome-wide association studies, and functional validations, thereby not only updating the MMD genetic landscape but also offering clinically relevant perspectives on the molecular pathogenesis of MMD and revealing emerging potential therapeutic targets of this increasingly recognized cerebrovascular disorder.

## 2. Literature Search Strategy

To conduct a comprehensive review, a literature search was performed using electronic databases including PubMed and Web of Science to identify relevant studies published between January 2022 and February 2025. The search strategy incorporated a combination of keywords, including “moyamoya disease”, “moyamoya syndrome”, “gene”, “genetic”, “epigenetic”, ”mutations”, “RNF213”, “p.R4810K”, ”angiogenesis”, ”vascular stenosis”, ”inflammation”, and “congenital diseases”. We supplemented the search by reviewing references from selected articles. The search was restricted to English-language publications. The final reference list was generated based on the relevance of each article to the review’s thematic focus. The graphic was designed and generated through Biorender.com.

## 3. RNF213 Gene

The *RNF213* gene, recognized as the predominant susceptibility gene for MMD, encodes an exceptionally large (591 kDa) multidomain protein (mysterin/RNF213) possessing bifunctional enzymatic properties: (i) AAA+ ATPase activity mediating mechanochemical transduction through oligomeric ring formation coupled with putative physical motion, and (ii) E3 ubiquitin ligase activity, mediating substrate ubiquitination for proteasomal degradation or signaling modulation [8]. Previous studies have also demonstrated that mysterin exhibits cell-autonomous antimicrobial activity [9,10]. Its expression is strongly upregulated by pro-inflammatory cytokines particularly in endothelial cells, macrophages, and fibroblasts [8,11]. Additionally, the dominant expression of mysterin eliminates adipose triglyceride lipase from lipid droplets and prevents fat mobilization [8]. These pleiotropic effects establish *RNF213* as a central hub integrating hemodynamic stress response, neuroinflammation, and metabolic adaptation in MMD pathogenesis.

Through its modulation of multiple signaling pathways, including the phosphoinositide 3-Kinase-AKT Serine/threonine kinase signaling cascade, matrix metalloproteinases (MMPs) activity, transforming growth factor β1(TGF-β1) signaling, basic fibroblast growth factor (bFGF) pathway, WNT/calcium/nuclear factor of activated T-cells 1 axis, and caveolin-1 system, the *RNF213* gene mutations lead to dysfunction of endothelial cells (ECs) and promote abnormal proliferation and migration of smooth muscle cells (SMCs), ultimately resulting in pathological angiogenesis [12,13,14,15,16]. Recent advances in in vitro experimental studies have provided substantial evidence supporting the pivotal role of *RNF213* in the regulation of angiogenic processes. Roy et al. established an innovative in vitro MMD model using CRISPR-Cas9-mediated *RNF213* gene knockout and illustrated an enhanced secretion of soluble pro-angiogenic factors and significant increase in angiogenesis in confluent ECs devoid of *RNF213* expression [13]. Findings from experimental studies utilizing *RNF213* knockdown in human umbilical vein endothelial cells have demonstrated that impaired angiogenesis is mediated, at least partially, through the downregulation of critical DNA replication and cellular proliferation pathways [17]. Furthermore, experimental evidence has indicated *RNF213* knockdown induces significant alterations in cytoskeletal organization and contractile function in SMCs [17]. These mechanistic insights into *RNF213*-mediated angiogenesis regulation provide us with a more profound understanding of the molecular pathophysiology underlying MMD.

*RNF213* plays a multifaceted role in vascular pathophysiology, particularly through its involvement in vascular stenosis mechanisms. Through the endothelial nitric oxide synthase (eNOS)-derived nitric oxide (NO) pathway, *RNF213* regulates vasodilation and the vascular remodeling process [12,15]. It also modulates the extracellular matrix dynamics by interacting with matrix metalloproteinases (MMPs) and tissue inhibitors of metalloproteinases (TIMPs) [15,16]. Recent investigations by Roy et al. demonstrated that *RNF213* knockout in brain endothelial cells induces distinct morphological alterations and increases blood–brain barrier permeability, highlighting its critical role in maintaining cerebral endothelial integrity in MMD [14]. Moreover, previous studies have shown that the E3 ligase module of *RNF213* enhances nuclear factor kappa-B (NF-κB) signaling pathways, thereby contributing to inflammation and immune responses in MMD [15,18,19]. Mutations in *RNF213* can also activate the nuclear factor of activated T-cells signaling in ECs, potentially leading to the development of pathological moyamoya vessels [5]. Some cytokines, such as tumor necrosis factor-α and interferon-γ, have been shown to activate *RNF213* transcription in ECs through the AKT/protein kinase R pathway and protein tyrosine phosphatase-1B (PTP1B) [5,11,19]. In vitro studies have further confirmed that *RNF213* can enhance the ECs sensitivity to inflammatory stimulation [17]. Recent immunological studies have revealed that ovabumin protein-pulsed dendritic cells with either *RNF213*-knockout or *RNF213* c.14576G  > A mutation-knockin modifications exhibited significant impairments in antigen processing machinery, including reduced antigen uptake capacity, compromised proteolytic activity, and decreased formation of endosomes and lysosomes, thereby suggesting the essential role of *RNF213* in regulating antigen uptake, processing and presentation [20].

The *p.R4810K* (p.Arg4810Lys, c.14429G > A) mutation in the *RNF213* gene is the most prevalent genetic variant associated with MMD, particularly in East Asian populations, where it accounts for about 80% of familial MMD cases [21]. However, in Western moyamoya populations, *RNF213* mutations are less frequent and more heterogeneous than in East Asians, with rare variants such as *p.R4810K* occasionally reported, suggesting different genetic contributions to disease pathogenesis across ethnicities [15,22]. The *p.R4810K* variant demonstrated a gene dosage effect in MMD pathogenesis, with homozygous carriers exhibiting substantially more severe clinical phenotypes and earlier disease onset compared to heterozygous individuals [4]. Mechanistically, many studies have shown that the *p.R4810K* variant plays a critical role in angiogenesis dysregulation of MMD. Hitomi et al. revealed that overexpression of *p.R4810K* downregulated securin, induced mitotic abnormalities and increased the risk of genomic instability, thereby suppressing angiogenic activity in induced pluripotent stem cell (iPSC)-derived vascular endothelial cells [23,24]. Further, *p.R4810K* has been shown to reduce ATPase activity, indicating its anti-angiogenic role via oligomer stabilization, which was confirmed in endothelial cells carrying the *p.R4757K* mutation (the ortholog of human *p.R4810K*) [25]. Recently, the human umbilical vein endothelial cells with *RNF213 p.R4810K* variant showed autophagy inhibition after exposure to oxygen-glucose deprivation, supporting the pivotal role of autophagy impairment caused by the *RNF213* variant in MMD-induced endothelial cell dysfunction [26]. In conclusion, although the exact molecular mechanisms underlying *RNF213* in MMD still remain to be fully elucidated, current evidence strongly supports its multifunctional role in disease development. Future research is expected to uncover additional pathways through which RNF213 contributes to MMD pathophysiology, potentially opening new avenues for therapeutic interventions.

## 4. Specific Mutations Related to MMD

**Figure 1:**
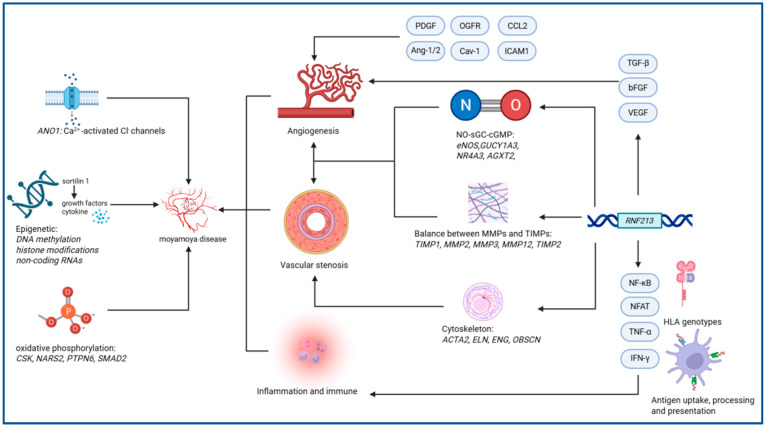
Integrated pathophysiological network of Moyamoya disease: Molecular crosstalk across angiogenic dysregulation, epigenetic modulators, and metabolic-immune axis. *Italicized* text indicates relevant genes; PDGF: platelet-derived growth factor; OGFR: opioid growth factor receptor; CCL2: cell chemokine ligand 2; Ang-1/2: angiopoietin-1/2; Cav-1: caveolin-1; ICAM1: intercellular adhesion molecule 1; TGF-β: transforming growth factor β; bFGF: basic fibroblast growth factor; VEGF: vascular endothelial growth factor; NF-κB: nuclear factor κ-B; NFAT: nuclear factor of activated T-cells; TNF-α: tumor necrosis factor-α; IFN-γ: interferon-γ; NO-sGC-cGMP: nitric oxide-soluble guanylyl cyclase-cyclic guanosine monophosphate; MMPs: matrix metalloproteinases; TIMPs: tissue inhibitors of metalloproteinases.

Specific mutations related to MMD refer to genetic biomarkers that have been identified in previous studies as having direct and specific correlations with MMD pathogenesis [6]. These genetic biomarkers demonstrate high sensitivity and specificity. Although the relationship between these specific mutations and MMD is complex, pathological and genetic studies have classified them into the following pathways: angiogenesis-related genes, vascular stenosis-related genes, inflammation and immune-related genes, and novel pathways proposed in recent years [5,6]. Notably, the majority of genes associated with MMD are not confined to a single pathogenic mechanism but rather participate in multiple pathways, with certain pathways demonstrating more significant contributions to disease pathogenesis than others. The interrelationships between various MMD-associated pathogenic pathways are systematically summarized in Figure 1.

### 4.1. Angiogenesis-Related Genes

Physiologic angiogenesis is a tightly regulated cascade involving vasodilation, endothelial cell migration and proliferation, lumen formation, endothelial survival, differentiation, and vascular remodeling [27]. The regulation of the angiogenesis process involves a complex interplay of growth factors and their signaling pathways, such as angiopoietin (Ang-1/Ang-2), vascular endothelial growth factor (VEGF), platelet-derived growth factor (PDGF), and TGF-β [4,28]. These molecules mediate endothelial cell proliferation, migration, and vascular stabilization through receptors such as VEGFR2, angiopoietin-1 receptor (Tie-2), and PDGFR [27]. MMD-associated genetic variants promote aberrant angiogenesis through dysregulated expression levels (upregulation or downregulation) or functional deficits in these critical regulatory factors. Extensive sequencing research has revealed distinct polymorphisms in various genes with differential expression in MMD populations, which affect growth factor expression and their receptor signaling pathways, including *TIE1*, *TIE2*, *ANGPT2*, *PENK*, *VEGF*, *PDGFRB*, *TGFB1*, *ICAM1*, *CXCL12,* and *CAV1* (summarized in Table 1) [29,30,31,32,33,34,35,36,37,38]. Recent investigations have expanded our understanding of angiogenesis-related genetic factors in MMD. Emerging evidence highlights the role of *RAPTOR*, a key regulator of hypoxia-inducible factor, in human leukocyte antigen class I antibody-mediated endothelial cell proliferation [39]. Notably, rare *RAPTOR* polymorphism may account for the disparate prevalence of MMD between East Asians and Caucasians [39]. The delicate equilibrium between MMPs and TIMPs, particularly MMP2 and TIMP2 expression, has been established as a critical regulator of angiogenesis [40,41]. Furthermore, the cell chemokine ligand 2 (CCL2) and its receptor (CCR2) signaling axis has been implicated in the pathogenesis of MMD through its modulating of the angiogenic responses of human endothelial cells [42]. Wang et al. performed whole exome sequencing and functional validation and discovered that *CAPN1* variants are a susceptibility gene of Chinese MMD, which is involved in the maintenance of vascular endothelial cell integrity and in the regulation of angiogenesis [43]. These findings suggest that MMD-associated aberrant angiogenesis results not only from growth factor-related gene alterations but also from disruptions in multiple pathways affecting ECs proliferation and vascular integrity.

### 4.2. Vascular Stenosis-Related Genes

The pathogenesis of vascular stenosis in MMD primarily involves concentric fibrocellular intimal hyperplasia, characterized by the proliferation and migration of ECs and SMCs, as well as extracellular matrix remodeling dominated by collagen deposition and elastin fragmentation, ultimately resulting in progressive intimal thickening and thus luminal occlusion [7]. Secondary to aberrant vascular anatomy, altered hemodynamics, pathological rheology, disturbances in wall shear stress, hypoxia and inflammatory responses, downstream mediating factors further modulate vascular proliferation and vasomotor tone at the molecular level, thereby contributing to stenotic progression in MMD [44,45]. Several genes have been reported to be involved in the above-mentioned mechanism via the various pathways leading to vascular stenosis in MMD. Genomic analyses have revealed significant differential expression of *OBSCN* (regulating obscurin-mediated cytoskeletal organization), *ACTA2* (α-smooth muscle actin polymerization), *ELN* (elastin fiber integrity), and *ENG* (endoglin-dependent TGF-β signaling), between the MMD patients and controls [31,46]. These genes regulate the expression and function of these key structural proteins, influencing the cellular morphology and cytoskeleton of SMCs and ECs, and thereby contributing to the pathogenesis of MMD [7,31,46,47,48]. The nitric oxide-soluble guanylyl cyclase-cyclic guanosine monophosphate (NO-sGC-cGMP) signaling pathway, curtail for vascular tone regulation [4,7], is frequently disrupted in MMD. Polymorphisms in *eNOS* primarily affect its expression and activity, while mutations in *GUCY1A3* impair NO-sensitive soluble guanylyl cyclase function (sGC, the major receptor for NO), both contributing to the pathogenesis in MMD [12,49,50]. Whole exome sequencing further implicates disrupted NO metabolism through *NOS*, *NR4A3* (hypoxia-responsive transcription), *ITGAV* (ECs adhesion), *GRB7* (signal transduction), and *AGXT2* (de novo variants altering asymmetric dimethylarginine catabolism), establishing a polygenic framework in which cytoskeletal dysregulation, extracellular matrix instability, and NO-cGMP pathway defects converge to drive stenotic progression [51].

The imbalance between MMPs and TIMPs, particularly involving *TIMP1*, *MMP2*, *MMP3*, *MMP12*, and *TIMP2*, can also disrupt the dynamics of ECs and SMCs and promote the pathological features of MMD [4,7,40,52]. Metabolic factors also contribute to vascular stenosis, with elevated homocysteine levels identified as a significant risk factor for vascular stenosis in MMD patients [53]. *MTHFR* and *TCN2*, which regulate homocysteine metabolism, have been implicated as susceptibility genes for MMD [54]. Apart from the above-mentioned pathways, several additional genes are recently recognized to be involved in the vascular stenosis of MMD. The overexpression of *circZXDC* (ZXD family zinc finger C) was found to upregulate *ABCC6*, which induces endoplasmic reticulum stress and subsequently promotes SMCs phenotypic switching from the contractile to synthetic states, ultimately contributing to the intimal thickening in MMD [55]. *HDAC9* is a member of a large family of genes that encode proteins responsible for the deacetylation of histones, thereby regulating chromatin structure and gene transcription [56]. Duan et al. identified *HDAC9* with genome-wide significance in MMD patients [54]. *CCL21*, *CEBPA*, *KRT18*, and *TNFRSF11A* were recently identified to be involved in endothelial-mesenchymal transition processes and thus contribute to the pathogenesis of MMD [57]. The *FOXM1* c.1205 C > A variant in unilateral MMD significantly attenuated the proangiogenic effects of the transcription factor forkhead box M1 in human brain endothelial cells, leading to reduced proliferation, migration, and tube formation [58]. This comprehensive genetic landscape underscores the complex interplay of multiple pathways in MMD vascular stenosis, highlighting potential targets for therapeutic intervention and personalized treatment strategies.

### 4.3. Inflammation and Immune-Related Genes

Although classic histopathology in patients with MMD did not reveal overt inflammatory changes [2], accumulating evidence suggests that dysregulated immune and inflammatory responses significantly contribute to intimal hyperplasia and pathological vascularization in MMD [5,59,60]. Previous research has highlighted that inflammatory responses may serve as a trigger for abnormal angiogenesis in MMD, with some immune factors such as NF-κB and interferons involved in the signal transduction of pathological moyamoya vessel formation [27]. Vacuolated degenerated SMCs were observed migrating into the thickened intima and exhibiting aberrant expression of IgG and S100A4, suggesting that the deposition of these immune-related factors promotes abnormal SMCs migration, thereby leading to intimal thickening and vascular stenosis [2,61]. Beyond the well-established role of *RNF213* in MMD-associated inflammation, several other inflammation and immune-related genes are also considered to be involved in the pathogenesis of MMD. Compared with the sera from healthy controls, six MMD-associated autoantibodies targeting APP, GPS1, STRA13, CTNNB1, ROR1, and EDIL3 were identified in the sera of MMD patients [62]. Human leukocyte antigen (HLA) genotypes demonstrate ethnic-specific associations with MMD, though their role as susceptibility factors remains inconclusive due to inconsistent study reproducibility [2,4]. The identification of a *CIAS1* gene mutation in a case of neonatal onset multisystem inflammatory disease with moyamoya syndrome highlights the potential involvement of interleukin-1β [63]. Furthermore, *UNC13D* was identified as a differentially expressed gene associated with neutrophil infiltration in MMD, demonstrating promising diagnostic specificity and sensitivity [60]. Twenty-eight key crosstalk genes, including *CAMP*, *NLRP12*, *CCL4*, *HLA-DRB5,* and *CD68*, were identified both in MMD patients and systemic lupus erythematous (SLE) patients, suggesting that the activation of T cells and monocyte-mediated immune responses play a significant role in the association between these two conditions [64]. Altogether, these findings underscore the complex interplay between inflammatory pathways and MMD pathogenesis.

### 4.4. Novel Pathways Proposed in Recent Years

With the recent advancements in the genetic exploration of MMD, novel pathways distinct from the aforementioned mechanisms also deserve attention. The application of genome-wide approaches to epigenomic analysis has progressively expanded the scope of observed pathological epigenetic alterations in MMD, and epigenetically dysregulated endothelial cells and smooth muscle cells proliferation, apoptosis, and migration represent a key pathogenic mechanism underlying intracranial arterial stenosis/occlusion and moyamoya vessel formation [65]. Accumulating evidence has revealed the important role of epigenetic mechanisms, including DNA methylation, histone modifications and non-coding RNAs networks, in regulating key cellular and molecular processes involved in the pathogenesis of MMD [65,66]. Previous studies have demonstrated that MMD patients exhibit sortilin 1 hypomethylation and upregulated expression in endothelial progenitor cells, which dysregulates angiogenic factors (elevating VEGF, VEGFR-1, bFGF, MMP9 while suppressing angiopoietin-1 and thrombospondin 2). This epigenetic alteration exhibited a positive correlation with proinflammatory cytokines (including C-reactive protein, Interleukin-6 and Interferon-γ), suggesting its dual role in vascular pathogenesis and potential as a clinical biomarker for MMD [65,67,68]. Impaired histone acetylation-mediated suppression of retinaldehyde dehydrogenase 2 may drive pathological moyamoya vasculogenesis [65,69]. Concurrently, de novo variants in histone modification and chromatin remodeling genes (*CHD4*, *CNOT3*, and *SETD5*) have been identified in the European MMD population [65,70]. As key members of the non-coding RNA family, microRNAs (miRNAs) have been implicated in MMD pathogenesis. Dai et al. demonstrated that dysregulated serum miRNAs collectively suppress RNF213 and BRCC3 protein expression at the post-transcriptional level, leading to impaired angiogenesis in MMD [71]. Additionally, elevated miRNA let-7c levels in MMD patients may also contribute to disease pathogenesis by targeting RNF213 [72]. Recently He et al. discovered key epigenetic regulators demonstrate spatial specificity: downregulation of *SOX6* and *RBM33* correlates with vascular occlusion, while the overexpression of *KCNMA1* and *GALNT2* appears to promote vascular hyperplasia responses to hemodynamic stress [73]. Crucially, genome-wide DNA methylation profiling conducted by Tokairin et al. revealed that patients with MMD exhibited decreased methylation variability at loci associated with critical biological processes, including methylation and transcription, DNA repair, cytoskeletal remodeling, natural killer cell signaling, and cellular migration [66]. These findings suggest epigenetic-immune crosstalk drives disease progression. Altogether, this pioneering research may not only advance our understanding of MMD pathogenesis but also facilitate the discovery of disease-specific biomarkers and therapeutic targets.

Genomic analyses incorporating pedigree reporting and DNA sequencing have expanded the repertoire of putative MMD susceptibility genes, with subsequent functional validation studies elucidating novel pathomechanisms beyond traditional angiogenic pathways. Notably, dysregulation of ion channel homeostasis has emerged as a key contributor in MMD pathophysiology. Ca^2+^-activated Cl^−^ channels are of importance in depolarization of vascular smooth muscle and contractile pericytes [74]. ANO1, encoding the Ca^2+^-activated Cl^−^ channel anoctamin-1, has been identified as a predisposing factor for MMD, with gain-of-function variants predisposing to involvement of the posterior circulation [75]. Furthermore, although the role of oxidative phosphorylation (OXPHOS) in MMD remains unclear, four genes involved in the OXPHOS pathway, *CSK*, *NARS2*, *PTPN6*, and *SMAD2*, were found to be differentially expressed between MMD patients and controls [76]. These genes are associated with angiogenesis, the proliferation of SMCs and ECs, and cytoskeleton regulation, suggesting their potential contribution to the pathogenic process of MMD [76]. These findings highlight the expanding spectrum of molecular mechanisms underlying MMD, offering new perspectives for understanding disease etiology and developing targeted therapeutic strategies.

## 5. Mutations Associated with Congenital Diseases Manifesting with MMS

MMS frequently coexists with several monogenic genetic disorders, suggesting shared molecular pathways in disease pathogenesis [2,3]. The underlying mechanisms involve several critical signaling pathways, including the Ras-Raf-mitogen activated protein kinase (MAPK) signaling pathway, the neurogenic locus notch homolog protein (Notch) signaling, and the genomic stability signaling pathway, as summarized in Table 2 [7]. Recently, whole exome sequencing studies have provided novel insights into the genetic architecture of MMS, revealing that its development results from the interplay between primary causative mutations and genetic modifiers [77]. Notably, specific modifier genes have been identified in different genetic contexts: *RNF213*, *MRVI1*, *BMPR2,* and *ABCC8* in neurofibromatosis type 1, and *RNF213*, *MRVI1,* and *NF1* in Down syndrome [77,78]. These findings strongly support the conceptual framework of MMD as a complex, multifactorial disorder resulting from the convergence of multiple genetic influences and molecular pathways.

### 5.1. Ras-Raf-MAPK Signaling Pathway

Dysregulation of the Ras-Raf-MAPK signaling cascade, a master regulator of cellular proliferation, apoptosis, and differentiation, underlies a spectrum of developmental disorders termed RASopathies, which exhibit strong associations with MMS [7,44]. In neurofibromatosis type 1, downregulation of neurofibromin expression due to loss of function mutations of *NF1* breaks down the Ras-Raf-MAPK signaling pathway and thus results in an enhanced mitotic signaling of ECs [79,80,96]. Meanwhile, the pathological consequences of neurofibromin deficiency induce the aberrant migration of SMCs to the intima and subsequent SMC proliferation [44]. Similarly, gain-of-function mutations in the *NRAS*, *SOS1*, *RAF1*, *BRAF*, *KRAS*, *PTPN1,* and *MAP2K1* genes observed in Noonan syndrome amplify MAPK/ERK signaling, characterized by a short and webbed neck, developmental delay, congenital cardiac defects, and skeletal abnormalities [7,81,82,86]. Costello syndrome arises from *HRAS* and *KRAS* mutations that disrupt the Ras-Raf-MAPK signaling pathway [7,85]. The sustained cellular proliferation driven by H-Ras protein, impaired elastogenesis due to reduced elastin gene expression, and elevated MMP-9 levels resulting from MAPK/ERK pathway activation are all potential contributing factors to this syndrome [44,97]. Of particular relevance to cerebrovascular pathology, mutations in *SPRED1* have been reported to cause Legius syndrome with MMS and the role of *SPRED1* in inhibiting ECs proliferation suggests that this may be responsible for the progressive cerebrovascular stenosis observed in this condition [83,84]. These findings collectively position Ras-Raf-MAPK dysregulation as a unifying mechanism across MMS-associated RASopathies, linking developmental signaling defects to cerebrovascular remodeling through convergent pathways of cellular hyperactivation, matrix instability, and hemodynamic stress potentiation.

### 5.2. Notch Signaling Pathway

The Notch signaling pathway serves as a critical regulator of vascular hemeostasis by suppressing ECs proliferation and migration, thereby maintaining angiogenic quiescence and stabilizing newly formed vessels through Delta-like ligand/Notch receptor cross-talk [98]. Alagille syndrome is a multisystem autosomal dominant disorder that serves as a major genetic etiology of moyamoya syndrome, primarily through pathogenic disruption of the Notch signaling pathway [6,7,87,88,99]. Approximately 90% of Alagille syndrome cases are caused by loss-of-function mutations in the *JAG1* gene (encoding Jagged-1 ligand), while 5–7% are attributable to *JAG1* gene deletions [6]. Endothelial-derived Jagged1 activates Notch signaling to drive adjacent vascular smooth muscle development, while Jagged1 deficiency impairs SMCs recruitment and differentiation, resulting in compromised vascular integrity [100]. Notably, a small subset of Alagille syndrome cases involves mutations in the NOTCH2 receptor, although these occurrences are relatively rare [6,88]. These genetic alterations lead to deficiency of Notch pathway components, resulting in characteristic cerebrovascular anomalies including progressive intracranial arterial stenosis and collateral vessel formation [87].

### 5.3. Genomic Stability Signaling Pathway

Microcephalic osteodysplastic primordial dwarfism type II (MOPD II) and Seckel syndrome, characterized by intrauterine growth restriction, microcephaly, and short stature, represent distinct genetic disorders converging on the genomic stability signaling pathway, with MOPD II arising from *PCNT* mutations and Seckel syndrome resulting from mutations in *CEP63*, *ATR*, *CENPJ*, *NIN*, *CEP152*, and *RBBP8* [6,7]. The developmental mismatch between cerebral growth and cerebrovascular maturation may serve as a triggering factor for moyamoya syndrome formation [44]. The genes mentioned above contribute to both primary genetic diseases and MMS through their critical roles in cell cycle progression and DNA repair mechanisms, and other aspects [7,89,90,91,92,101]. Expanding the spectrum of genomic instability-related disorders, CHOPS syndrome caused by *AFF4* mutations has been reported to share similar pathogenic mechanisms [93]. Furthermore, the chromosome Xq28 region harbors several genes implicated in DNA repair and phosphorylation processes. Notably, *BRCC3* mutations underlie severe hemophilia A and moyamoya (SHAM) syndrome, while *MECP2* duplications are responsible for *MECP2* duplication syndrome, both demonstrating the critical role of X-linked genomic stability in cerebrovascular pathology [94,95,102,103].

### 5.4. Other Unclassified Pathways

The expanding spectrum of moyamoya syndrome (MMS)-associated genetic disorders reveals diverse pathomechanisms converging on cerebrovascular remodeling, extending beyond canonical pathways to encompass hematologic, metabolic, and mitochondrial pathologies. Sickle cell disease (SCD), caused by homozygous mutations in the *HBB* gene encoding β-globin, demonstrates a particularly strong association with MMS-like vasculopathy. The pathophysiology involves multiple mechanisms, including abnormal sickle-shaped erythrocytes causing chronic hemolysis and endothelial damage, increased blood viscosity impairing cerebral perfusion, and recurrent vaso-occlusive crises triggering hypoxic stress [6,104]. These factors collectively promote pathological vascular remodeling characterized by intimal hyperplasia and neovascularization, closely resembling the stenotic-occlusive lesions and collateral vessel formation seen in MMD. In Down syndrome, the high prevalence of MMS suggests potential interactions between the *RNF213* gene and the genes on chromosome 21 regulating vascular physiology and elasticity in these patients [105]. For Down syndrome patients with MMS, elevated endostatin levels and consequent inhibition of angiogenesis appear to stimulate premature vascular branching, resulting in pathological collateral networks that mimic the characteristic moyamoya vessels [106]. The spectrum of MMS-associated disorders extends to immune dysregulation syndromes, as exemplified by Aicardi-Goutières syndrome type 5, where *SAMHD1* mutations impair innate immune response through selectively inhibiting activation of the NF-κB and interferon-1 pathways in cells [107,108]. Interestingly, a case of multiple endocrine neoplasia type 2A (MEN2A) with concurrent *RNF213* and *RET* mutations demonstrated potential synergistic effects between genetic predisposition and catecholamine hypersecretion in intracranial stenosis development [109]. Mitochondrial dysfunction has emerged as another pathogenic mechanism, with the *TOMM7* variant leading to microcephalic osteodysplastic dwarfism with MMS, underscoring the importance of mitochondrial function and energy metabolism [110]. Glycogen storage disease type I, an autosomal recessive disorder caused by glucose-6-phosphatase deficiency, manifests with life-threatening hypoglycemia and progressive hepatorenal pathology, demonstrating a significant clinical association with MMS [111]. Hong et al. postulated that *RNF213* variants in certain patients may interact with metabolic dysfunction, potentially driving progressive steno-occlusive arteriopathy in glycogen storage disease type I [112]. However, the genetic interplay between these disorders requires further investigation. Although some other inherited metabolic diseases such as 17a-hydroxylase/17,20-lyase deficiency and D-2-hydroxyglutaric aciduria have been also associated with MMS, suggesting a broader metabolic-vascular crosstalk, their mechanistic links to cerebrovascular pathology remain to be elucidated [113,114]. This heterogeneity underscores MMS as a final common pathway for disparate genetic insults converging on endothelial-hematopoietic-mesenchymal axis dysregulation.

## 6. Discussion and Perspectives

MMD represents a complex cerebrovascular disorder with multifactorial genetic underpinnings, involving dysregulation of multiple interconnected pathways including angiogenesis, vascular remodeling, and inflammatory responses. In contrast, MMS typically predominantly manifests as a secondary vasculopathy within monogenic disease frameworks, where primary genetic defects (e.g., *JAG1* in Alagille syndrome, *NF1* in RASopathies) disrupt cerebrovascular homeostasis through pathway-specific mechanisms. The recognition of these dual genetic mechanisms highlights the necessity for a comprehensive research strategy that combines systems-level multi-omics analyses to establish genotype-phenotype relationships, experimental validation of candidate genes using disease-specific models, and the development of comprehensive and appropriate analytical approaches that simultaneously target both initiating factors and their downstream pathological consequences. Emerging technologies such as CRISPR/Cas9 gene editing and single-cell RNA sequencing (scRNA-seq) provide powerful tools to unravel the molecular pathogenesis of MMD. The CRISPR/Cas9 platform enables precise functional characterization of disease-associated variants in genes such as *RNF213* and *GUCY1A3* through isogenic cell line engineering, facilitating mechanistic investigations into their contributions to vascular smooth muscle dysfunction and angiogenic impairment [14,115]. Meanwhile, scRNA-seq provides unprecedented resolution to map cellular heterogeneity within moyamoya vessels, identifying distinct pathogenic endothelial, smooth muscle, and immune cell subpopulations [116,117]. Integration of these approaches can delineate cell-type-specific molecular networks underlying characteristic intracranial stenosis and collateral formation. These methodological advancements collectively bridge critical knowledge gaps between genetic associations and functional pathophysiology, laying the foundation for a mechanism-driven therapeutic development in MMD.

## Figures and Tables

**Table 1:** Growth factor-related genes.

Chromosome	Gene	Related Growth Factor	Study Type	Biomarker Validation Status
Chromosome 1	*TIE1* [35,38]	angiopoietin-1 and angiopoietin-2	functional analyses	in vitro
Chromosome 9	*TIE2* [35,36]	angiopoietin-1 and angiopoietin-2	functional analyses	in vitro and clinical
Chromosome 8	*ANGPT2* [38]	angiopoietin-2	functional analyses	in vitro
Chromosome 8	*PENK* [34]	opioid growth factor receptor and delta opioid receptor	functional analyses	in vitro
Chromosome 6	*VEGF* [32]	vascular endothelial growth factor	case-control	clinical
Chromosome 22	*PDGFRB* [30]	platelet-derived growth factor	case-control	clinical
Chromosome 19	*TGFB1* [30]	transforming growth factor β	case-control	clinical
Chromosome 19	*ICAM1* [37]	intercellular adhesion molecule 1	case-control	in vitro
Chromosome 10	*CXCL12* [31]	C-X-C motif chemokines and vascular endothelial growth factor	case-control	clinical
Chromosome 7	*CAV1* [33]	Caveolin-1	case-control	in vitro and clinical

**Table 2:** Different pathways involving the pathogenic genes of primary genetic diseases with MMS.

Pathway	Primary Genetic Disorder	Chromosome	Gene
Ras-Raf-MAPK signaling pathway	neurofibromatosis type 1 [79,80]	17q11.2	*NF1*
	Noonan syndrome [81,82]	1p13.2	*NRAS*
		2p22.1	*SOS1*
		3p25.2	*RAF1*
		7q34	*BRAF*
		11q23.3	*CBL*
		12p12.1	*KRAS*
		12q24.13	*PTPN11*
		15q22.31	*MAP2K1*
	Legius syndrome [83,84]	15q14	*SPRED1*
	Costello syndrome [85,86]	11p15.5	*HRAS*
		12p12.1	*KRAS*
Notch signaling pathway	Alagille syndrome [87,88]	1p12-p11	*NOTCH2*
		20p12.2	*JAG1*
Genomic stability signaling pathway	Seckel syndrome [89,90]	3q23	*ATR*
		3q22.2	*CEP63*
		13q12.12-q12.13	*CENPJ*
		14q22.1	*NIN*
		15q21.1	*CEP152*
		18q11.2	*RBBP8*
	Microcephalic Osteodysplastic Primordial Dwarfism Type II [91,92]	21q22.3	*PCNT*
	CHOPS syndrome [93]	5q31.1	*AFF4*
	Severe hemophilia A and moyamoya (SHAM) syndrome [94]	Xq28	*F8, BRCC3*
	MECP2 duplication syndrome [95]		*MECP2*

## Data Availability

Not applicable.
